# Alu elements shape the primate transcriptome by *cis*-regulation of RNA editing

**DOI:** 10.1186/gb-2014-15-2-r28

**Published:** 2014-02-03

**Authors:** Chammiran Daniel, Gilad Silberberg, Mikaela Behm, Marie Öhman

**Affiliations:** 1 Department of Molecular Biosciences, The Wenner-Gren Institute, Stockholm University, Stockholm, Sweden; 2 Present address: Unit of Computational Medicine, Center for Molecular Medicine, Karolinska Institute, Karolinska University Hospital, Stockholm, Sweden

## Abstract

**Background:**

RNA editing by adenosine to inosine deamination is a widespread phenomenon, particularly frequent in the human transcriptome, largely due to the presence of inverted Alu repeats and their ability to form double-stranded structures – a requisite for ADAR editing. While several hundred thousand editing sites have been identified within these primate-specific repeats, the function of Alu-editing has yet to be elucidated.

**Results:**

We show that inverted Alu repeats, expressed in the primate brain, can induce site-selective editing in *cis* on sites located several hundred nucleotides from the Alu elements. Furthermore, a computational analysis, based on available RNA-seq data, finds that site-selective editing occurs significantly closer to edited Alu elements than expected. These targets are poorly edited upon deletion of the editing inducers, as well as in homologous transcripts from organisms lacking Alus. Sequences surrounding sites near edited Alus in UTRs, have been subjected to a lesser extent of evolutionary selection than those far from edited Alus, indicating that their editing generally depends on *cis*-acting Alus. Interestingly, we find an enrichment of primate-specific editing within encoded sequence or the UTRs of zinc finger-containing transcription factors.

**Conclusions:**

We propose a model whereby primate-specific editing is induced by adjacent Alu elements that function as recruitment elements for the ADAR editing enzymes. The enrichment of site-selective editing with potentially functional consequences on the expression of transcription factors indicates that editing contributes more profoundly to the transcriptomic regulation and repertoire in primates than previously thought.

## Background

RNA editing by adenosine deamination is a co- or post-transcriptional alteration of mRNA as well as non-coding RNA, which occurs in metazoans. This adenosine to inosine (A-to-I) editing occurs at single adenosines in transcripts produced by RNA polymerase II. A-to-I RNA editing within double-stranded RNA (dsRNA) is catalyzed by the ADAR family of enzymes (ADAR1 and ADAR2) [1], proven to have catalytic activity on a number of transcripts mostly expressed in the brain (reviewed in [2]). The frequency of edited sites in an ADAR substrate usually increases with the length and double-strandedness of the duplex [3].

Selective editing of single adenosines is often found in short duplexes interrupted by bulges and internal loops. Since inosine is interpreted as a guanosine by the cellular machinery, editing has the potential to recode an mRNA and thereby increase the protein repertoire. In mammals most transcripts subjected to site-selective A-to-I editing within a coding sequence have been found in genes involved in neurotransmission. ADAR-mediated site-selective editing leads to altered functionality of several ligand- and voltage-gated ion channels as well as G-protein-coupled receptors in the mammalian brain [4-9]. One of the brain-specific edited transcripts codes for the α3 subunit in the GABA_A_ receptor (Gabra-3), where editing (by either ADAR1 or ADAR2) recodes an isoleucine to a methionine (I/M) [10]. We recently showed that an individual long intronic hairpin structure located 150 nucleotides downstream of the hairpin including the I/M site in Gabra-3 is required for efficient editing [11]. Although this editing inducer element (IE) is hyper-edited, mutational analysis shows that it is the double-stranded structure rather than editing that is important for the distal editing induction. These results indicate that this *cis*-acting structural element, downstream of the sequence required for A-to-I catalysis, increases the local concentration of the editing enzyme by attracting ADAR, thus enabling editing in the vicinity.

The repetitive retrotransposable Alu elements, each spanning approximately 300 nucleotides, are abundantly interspersed throughout the primate genome and are present in approximately 75% of all human genes, mostly within introns and untranslated regions (UTRs). Adjacent inverted Alu repeats can pair and form long stable stem-loop structures, which are favorable editing substrates, and are also potentially highly abundant in humans (there are 228,607 inverted Alu pairs within 1 kb in genes from the NCBI Reference Sequence (RefSeq) database). The human transcriptome is therefore exceptionally prone to A-to-I editing in comparison to other mammals and even to other primates, which is mainly attributed to the high frequency of editing within inverted Alu pairs [12]. Even compared to other repetitive elements, the primate-specific Alu repeats are particularly prone to editing, which occurs at multiple sites – a phenomenon specifically referred to as ‘hyper-editing’ [13-17]. Recent high-throughput analyses have revealed more than 500,000 A-to-I edited sites within human Alu repeats [15,18-20]. Interestingly, one of these analyses has also identified an enrichment of non-Alu editing events in the vicinity of edited Alus [20]. While this observation suggests an association between the two, neither direct evidence nor an underlying mechanism have been found.

Our results indicate that inverted Alu repeat elements can act as editing inducers. These elements are often located hundreds of nucleotides away from the specific editing site. We propose that duplexed inverted Alu repeats act as ADAR recruitment elements, which enhance editing efficiency at adjacent sites, ultimately giving rise to new editing events in primate transcriptomes.

## Results

### The intronic editing inducer element in Gabra-3 is independent of position

We have previously shown that a long intronic stem-loop structure, located 150 nucleotides downstream of the I/M editing site in the Gabra-3 transcript, is required for its editing, and is also targeted for editing itself [11]. In the presence of this intronic inducer, exonic I/M editing is efficient even in a short double-stranded structure, which cannot be edited independently.

**Figure 1:**
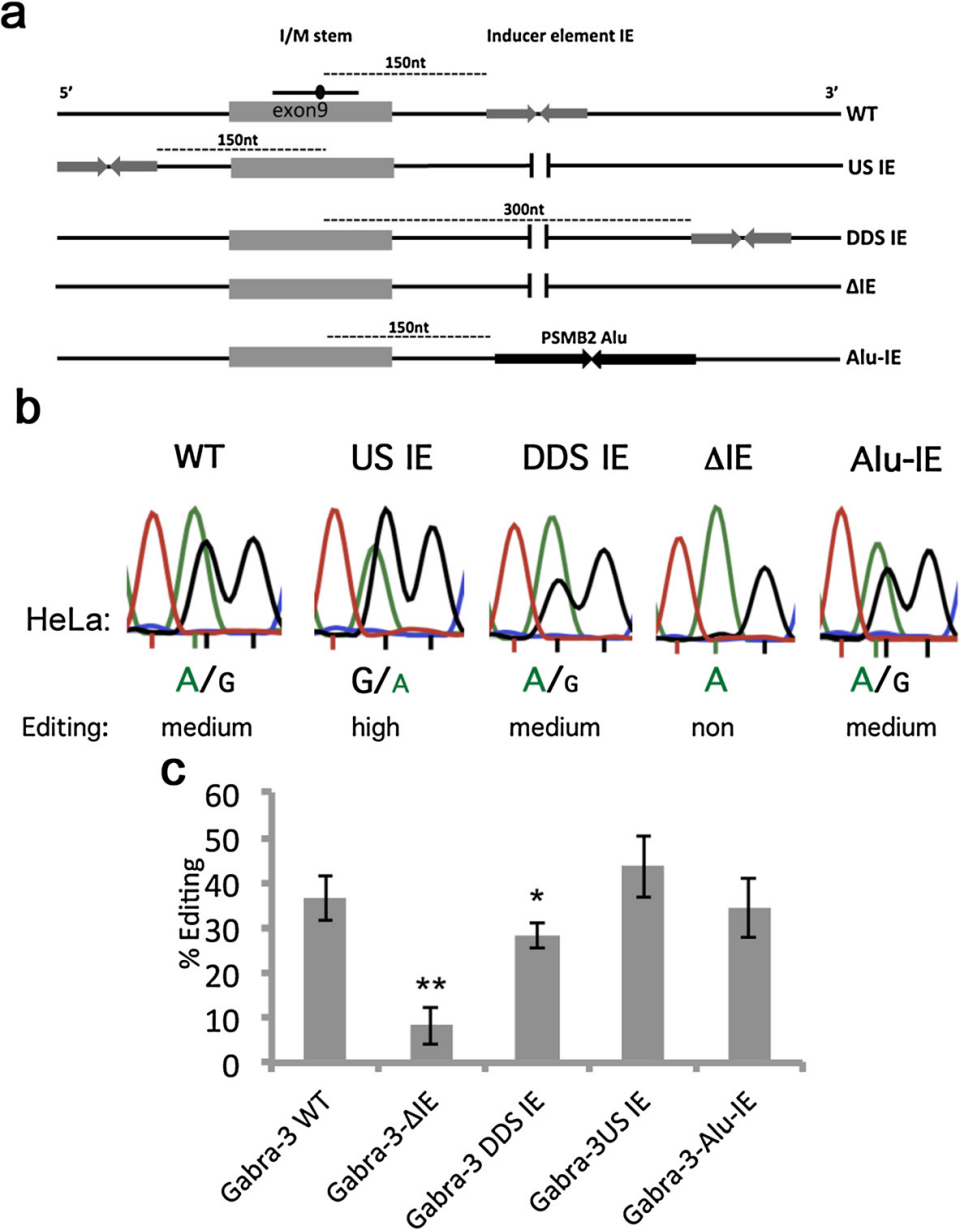
**Analysis of editing efficiency at the I/M site in Gabra-3 editing reporters in HeLa cells. (a)** Mouse Gabra-3 mutants used to analyze editing efficiency depending on the location of the inducer element. The I/M site is located in exon 9 of the Gabra-3 transcript and the dsRNA structure and editing site is illustrated as a line and a dot. The reverse arrows illustrate the position of the IE in the different mutants. In the WT construct the IE is positioned 150 nucleotides from the I/M site and illustrated as a dotted line. In the ΔIE mutant the IE is deleted. In the DDS IE mutant the IE is moved 300 nucleotides downstream of the I/M site and in the US IE the IE is moved 150 nucleotides upstream of the I/M site. In the Alu-IE, the native IE is replaced by the human inverted Alu found in the 3' UTR of the *PSMB2* gene. **(b)** Example Sanger sequence chromatograms of the I/M site after RT-PCR from transfections with Gabra-3 mutants. Editing is seen as a dual A and G peak. Below, reproducible triplicates were compared with known levels of I/M site editing using 454 high-throughput sequencing (see Materials and methods and [11]) and classified into different levels of editing from non to full. **(c)** Quantification of editing efficiency of the different Gabra-3 mutants. All mutants were tested at least in triplicate. The amount of edited transcript was determined by measuring the ratio between the A and G peak heights and represented as a percentage. The bars represent the mean value of the ratio between the A and G peak heights. Error bars are standard deviation. Significance: *P = 0.05, **P < 0.05 (two-tailed Student’s *t*-test) (for details see Materials and methods section). DDS IE, downstream inducer element; ΔIE, deleted inducer element; IE, inducer element; I/M, isoleucine to methionine; nt nucleotide; RT-PCR, reverse transcription polymerase chain reaction; US IE, upstream inducer element; WT, wild-type.

We wanted to analyze further how this IE is linked to I/M editing and determine whether the elements directly interact with each other. To investigate this, a construct containing the mouse wild-type (WT) *gabra-3* minigene, which includes the I/M site in exon 9 followed by the intron 9 editing inducer stem-loop, was analyzed (Figure 1a). The efficiency of editing at the I/M site was then compared to editing from constructs where the IE had been moved upstream of the I/M site in exon 9 (US IE), further downstream (DDS IE) of its original location or deleted (ΔIE) (Figure 1a). The reporters were transfected into HeLa cells expressing endogenously active ADAR. Also they were co-transfected with ADAR1 or ADAR2 expression vectors into HEK293 cells. To determine the editing efficiency at the I/M site, we used Sanger sequencing after RT-PCR on the extracted total RNA and measured the ratio between the A and G peak heights (for details, see Materials and methods and [11]).

As previously shown, no editing was detected by endogenous ADAR in HeLa cells when expressing Gabra-3 lacking the intronic inducer (ΔIE), while the WT transcript was edited to 37% (*P* = 0.001) (Figure 1a,b,c) [11]. Endogenous ADAR editing of the WT reporter was lower than when ADAR was transiently expressed from a vector, probably due to a lower concentration of the ADAR protein. Noteworthy is that editing at the I/M site then is totally dependent on the intronic inducer. Decreased I/M editing of the ΔIE transcript compared to WT was also observed in HEK293 cells after ADAR1 or ADAR2 co-transfection (data not shown). The efficiency of I/M editing when the inducer was placed 150 nucleotides upstream of the I/M site (US IE) as well as when the distance between the I/M site and the IE was increased to double the distance downstream (DDS IE) of its original location was comparable with the level of editing in the WT reporter (Figure 1b,c). Interestingly, I/M site editing in the US IE was even more efficient (45%) than in the wild-type reporter (37%, *P* = 0.20), although the difference was not statistically significant. A slight decrease in editing efficiency (34%, *P* = 0.03) could be detected when the editing inducer was moved to a double distance downstream of the I/M site (DDS IE). These results suggest that the location of the IE does not affect editing efficiency at the I/M site if it is in the vicinity of the site of editing.

### Editing induction is sequence independent and can be induced by Alu elements

We then wondered whether any long double-stranded structure can act as an editing inducer. To test if inverted Alu repeats, forming long stem-loops, can act as IEs and increase editing efficiency, we replaced the native Gabra-3 editing inducer with the inverted Alu elements from the 3′ UTR of the human *PSMB2* mRNA (Alu-IE) (Figure 1a). These inverted Alu repeats have previously been shown to be subjected to editing [21,22]. Indeed, when transfected into HeLa and HEK293 cells, the *PSMB2* Alus induced I/M editing to the same extent (*P* = 0.65) as the WT intronic IE (Figure 1b,c). These results indicate that inverted Alu repeats have the potential to act as editing inducers and that the induction of editing at the I/M site by the IE is sequence independent.

### Enrichment of selectively edited sites near edited Alu repeats in humans

We hypothesized that enhancement of site-selective editing by proximal inverted Alu repeats is widespread in the human transcriptome. To examine the relation between inverted Alu repeats and site-selective editing systematically, two datasets were compiled by literature mining (Table S1 in Additional file 1): one of non-Alu editing sites, and another of edited Alu elements. Several conservative filtering criteria were used to minimize the likelihood of false positives or experimental artifacts. We included only sites on RefSeq transcripts, and sites identified in tissues (rather than immortalized cells). In addition, only edited Alus were selected for analysis because they bear direct evidence for the presence of ADAR. However, it should be noted regarding the last criterion that editing of Alus is not a prerequisite of our hypothesis of ADAR recruitment, as ADAR binding does not always result in editing [23]. Thus, 10,650 non-Alu sites and 108,838 edited Alus (78.3% of which had inverted Alu within 1 kb) were selected (Table S2 in Additional file 2).

**Figure 2:**
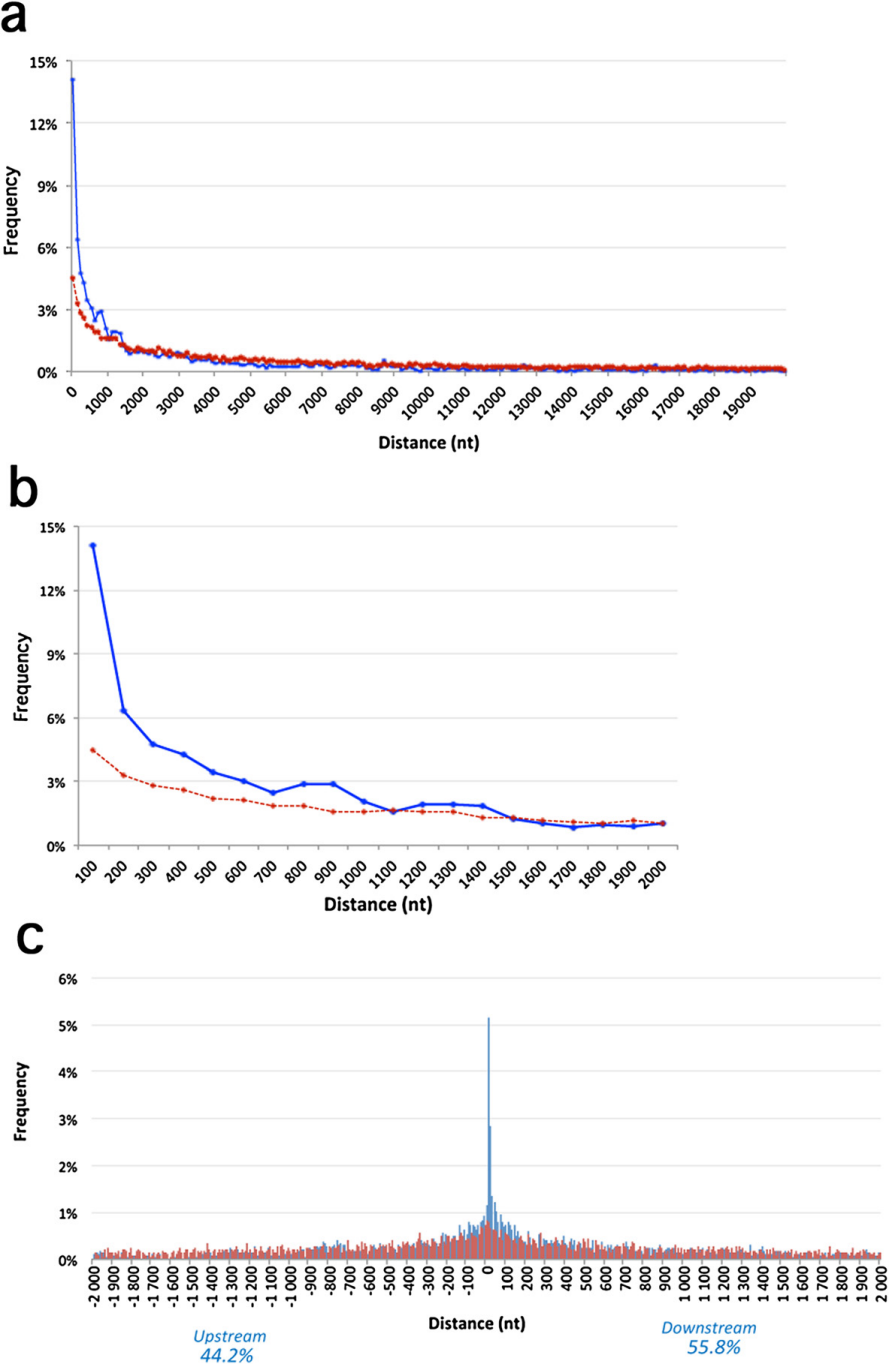
**Distance distribution between non-Alu editing sites and nearest edited Alu.** The distance from random adenosines is shown as a broken red line. The distances were grouped in bins of 100, and their frequencies were plotted for a distance window of **(a)** 20 kb and **(b)** 2 kb. **(c)** Distance distribution plot with orientation, in bins of 10 nucleotides. Positive and negative distances indicate that the edited Alu is downstream or upstream of the (non-Alu) editing site. Blue bars: distance from editing sites to edited Alus. Red bars: distance from random adenosines to edited Alus. A significant tendency for Alus to be located downstream was observed (*P* = 3 × 10^-16^). kb, kilobase; nt, nucleotide.

To examine whether the non-Alu sites were located closer to edited Alus than randomly expected, we selected 20,000 adenosine residues outside Alu elements. Then, we calculated the distances from each non-Alu editing site to the nearest edited Alu, and similarly between random adenosines and the nearest edited Alu (Figure 2a). Non-Alu editing sites were found to be significantly closer to edited Alus than random adenosines (*t*-test *P* < 3 × 10^-120^). Notably, this difference was due to the high frequency of editing sites located in close proximity (up to 1 to 2 kb) from an edited Alu (Figure 2b and Table S2 in Additional file 2). Interestingly, the nearest edited Alu was significantly more often located downstream (55.8%) than upstream (44.2%, *P* < 3 × 10^-16^) of these sites (up to 2 kb from the edited Alu) (Figure 2c). The depletion of edited sites downstream of edited Alus may be because Alu elements are frequently located in 3′ UTRs (approximately 10% in our data), whose full length is often obscured in gene model databases [24].

Another aspect that ought to be controlled for is the occurrence of non-Alu editing on a long stable duplex structure, which may be sufficient to recruit ADAR efficiently without assistance from other *cis*-elements. These cases are characterized by clustered editing, where multiple sites are edited within close proximity. Therefore, to focus on sites that are most likely located in short duplexes (such as the I/M site of Gabra-3), we removed editing clusters from the set of non-Alu sites. A cluster was defined as a group of at least three sites that are located up to 40 nucleotides from each other over a minimum total length of 70 nucleotides (from first site to last site). Thus, 1,312 sites were removed in total (Table S2, last column, in Additional file 2). When the distance analysis was repeated, similar results were observed, with a slightly increased enrichment for non-Alu sites in close proximity to edited Alus (Figure S1 in Additional file 3).

Altogether, our results show that non-Alu sites are significantly closer to edited Alus than random, clearly indicating an underlying mechanism that associates the two.

### Alu-dependent editing in UTRs is flanked by less conserved sequences than other edited sites

**Figure 3:**
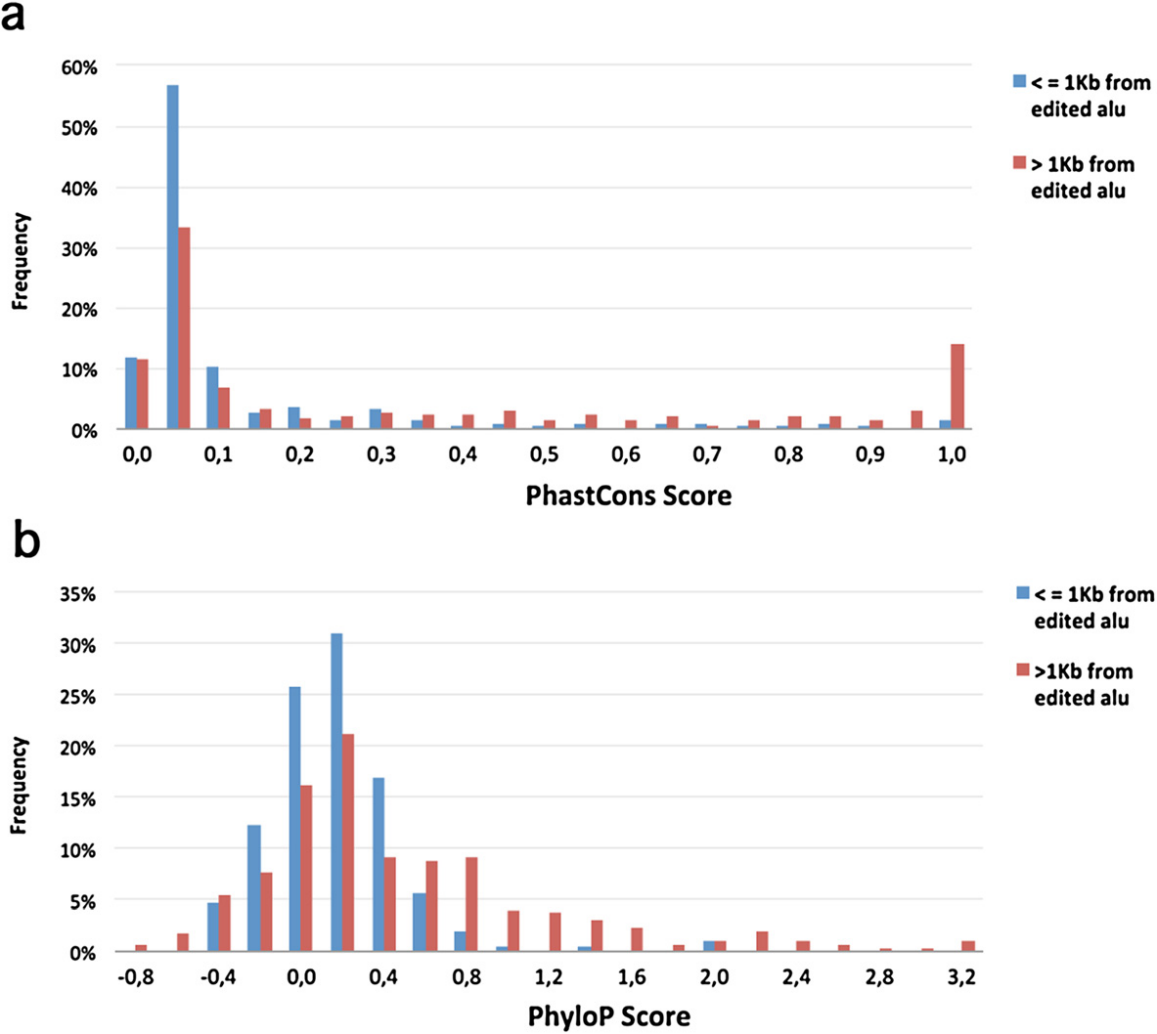
**Conservation of sequences flanking UTR editing sites proximal to (<=1 kb, blue) and distal to (>1 kb, red) edited Alu.** PhastCons **(a)** and PhyloP **(b)** scores for the editing site and 15 nucleotides upstream and downstream were averaged. A distinct group of distal sites within ultra-conserved elements can be observed ((a), highest score bin). kb, kilobase; UTR, untranslated region.

Since the editing of Alu-independent sites requires a highly stable and specific stem-loop structure, their flanking sequences have likely been subjected to stringent evolutionary selection. This may not be the case, however, for Alu-dependent editing sites, if their editing efficiency relies on external stem-loop structures (inverted Alu elements). To compare the conservation between these two groups of sequences (near and far from edited Alus), we collected non-clustered editing sites, which are most likely to have functional effects. These were sites leading to non-synonymous recoding of a protein and sites in the UTRs. For each editing site, the average conservation was calculated over the site and 30 flanking nucleotides, 15 from each side, using both PhyloP [25] and PhastCons [26] scores for 46 vertebrates. PhyloP evaluates signatures of selection at particular bases, whereas PhastCons takes into account conservation at neighboring sites, allowing detection of conserved elements. When non-synonymous sites were compared, no significant difference was observed between sequences flanking distal sites (*n* = 255) and those flanking proximal sites (*n* = 57; *t*-test *P* = 0.53 and Mann–Whitney U-test *P* = 0.36 for PhyloP and PhastCons, respectively). However, when sites in UTRs were compared, sequences flanking the distal sites (*n* = 213) were found to be far more conserved than those flanking the sites proximal to inverted Alu repeats (*n* = 297). See Figure S2 in Additional file 3. Interestingly, when the distribution of the conservation scores was examined, the PhastCons scoring scheme revealed a group of ultra-conserved sequences, which was not detected by PhyloP. Since PhastCons also estimates the probability that each nucleotide belongs to a conserved element, based on conservation at neighboring sites, this indicates that these sequences were conserved as structural modules rather than as individual sites (Figure 3). A detailed list of these sites is provided in Table S3 in Additional file 4. This observation demonstrates the scale of the phenomenon, where recently integrated Alu elements give rise to new editing events that otherwise would have still been dormant.

### Gene ontology enrichment analysis of genes with functional editing near edited Alus

Functional enrichment analysis was performed on genes containing editing sites within 1 kb of an edited Alu (Alu-dependent editing) that are likely to affect functionality. Some of these editing events lead to missense and nonsense recoding and splice signals (*n* = 245). To identify annotations for which genes with Alu-dependent editing specifically are enriched, we included all genes containing ‘functional’ sites of editing as a background (*n* = 1,113). In addition, to avoid detecting terms and functions enriched in Alu-containing genes, simply because all the examined genes have Alus, we added all Alu-containing genes to the background list (*n* = 16,545). The results revealed marked enrichment of functions such as transcription regulation (2.2-fold) and the lysosome pathway (46-fold). In agreement with the enrichment found for transcription regulation, high enrichment was observed for genes encoding zinc finger proteins and specific sub-families of these (up to 16-fold), which often function as transcription factors. Table 1 shows selected top categories, sorted by fold-enrichment. An enrichment of genes with multiple splice variants was also found, as well as expression in several tissues (uterus: 4.5-fold, placenta: 4.1-fold, testis: 2.4-fold, epithelium: 3.6-fold, skin: 3.3-fold and brain: 2-fold). It is noteworthy that while the fold-enrichment for high brain expression is more modest than for several other tissues, the frequency of brain-enriched genes is far higher than for any other tissue (44%). A complete list of all significantly enriched terms and genes is provided in Table S4 in Additional file 5.

**Table 1:** Selected top categories enriched in genes containing editing sites near an edited Alu

**Category**	**Term**	**Count**	**Percentage**^**a**^	***P *** **value**	**Fold enrichment**	**Benjamini**^**b**^	**False discovery rate**
KEGG_PATHWAY	hsa04142; lysosome	7	2.9	9.32 × 10^-9^	46.1	8.02 × 10^-7^	1.01 × 10^-5^
PFAM	PF01352; KRAB box (Krueppel-associated box)	15	6.2	9.97 × 10^-13^	16.0	4.71 × 10^-10^	1.42 × 10^-9^
PANTHER_FAMILY	PTHR23224; zinc finger proteins	17	6.9	1.94 × 10^-9^	7.3	3.57 × 10^-7^	2.40 × 10^-6^
INTERPRO	IPR013087; zinc finger, C2H2-type/integrase, DNA-binding	17	6.9	3.22 × 10^-8^	6.0	7.15 × 10^-6^	4.56 × 10^-5^
SP_PIR_KEYWORDS	Alternative splicing	111	45.3	5.35 × 10^-31^	3.1	7.24 × 10^-29^	7.03 × 10^-28^
GOTERM_BP_ALL	GO:0045449; regulation of transcription	43	17.5	8.91 × 10^-7^	2.2	5.22 × 10^-4^	0.0014

^a^Percentage of genes with the term of total genes in analyzed list; ^b^Benjamini–Hochberg corrected *P* value for multiple comparisons.

To unify similar annotation terms, we performed functional annotation clustering analysis with DAVID [27,28], which identified three enriched clusters (Table S5 in Additional file 6). The most significant, and abundant in annotations, contained zinc finger annotations, predominantly of the C2H2 family. This enrichment is likely to occur due to the abundance of Alu elements, which have integrated into zinc finger genes [20,29]. The two other clusters included terms related to the nuclear compartments and intracellular protein transport. Altogether, this indicates that Alu-dependent editing is involved in fundamental cellular processes and has the potential to exert a significant effect.

### Recoding of NEIL1 by editing is primate specific due to upstream Alu inverted repeats

**Figure 4:**
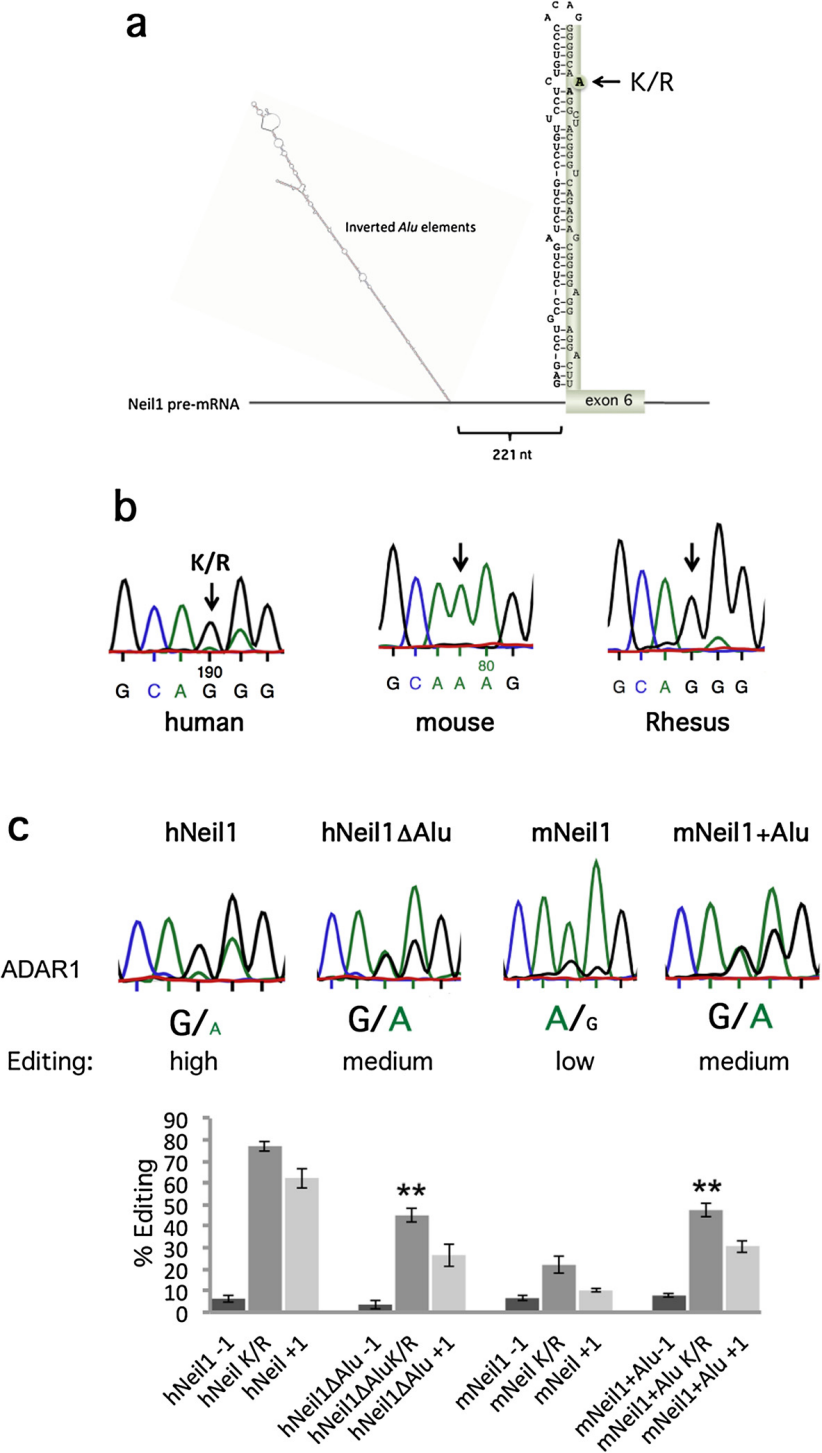
**Editing of the DNA repair enzyme NEIL1 *in vivo *and co-transfected in HEK293 cells. (a)** Intron 5 and exon 6 of human NEIL1 pre-mRNA. Two inverted *Alu* repeats located 200 nucleotides from the K/R stem are illustrated when it is folded using Mfold. The −1, K/R, +1 site found at the 5' end of exon 6 is highlighted. **(b)** Sanger sequencing chromatograms after RT-PCR of NEIL1 transcripts from human, mouse and rhesus brains. Editing was detected at the −1, K/R and +1 site in human and rhesus brains as a dual A and G peak. No editing was detected in the RNA from a mouse brain. **(c)** Top: Sequencing chromatograms after RT-PCR on RNA from co-transfections of ADAR1 with the human NEIL1 construct including the inverted *Alu* repeats (hNEIL1), inverted *Alu* repeats deleted (hNEIL1 ΔAlu), mouse NEIL1 (mNEIL1) and mNEIL1 where the inverted Alus from the human sequence were fused into the mouse sequence 200 nucleotides upstream of the K/R site (mNEIL1 + Alu). Bottom: Quantification of editing efficiency of the different NEIL1 constructs co-transfected with ADAR1 in HEK293 cells. Editing efficiency was calculated at the −1, K/R and +1 site. The mean value of the ratio between the A and G peak heights from at least three individual experiments were calculated as percentage editing. Error bars are standard deviation. Significance: *P = 0.05, **P < 0.05 (two-tailed Student’s *t*-test) (for details see Materials and methods section). K/R, lysine-to-arginine; nt, nucleotide; RT-PCR, reverse transcription polymerase chain reaction.

One of the genes that harbor site-selective editing in the vicinity of an edited Alu encodes the NEIL1 DNA repair enzyme. NEIL1 has previously been shown to be edited at the second and third positions of the K242 AAA codon within exon 6 [30,31]. Editing at the central position of the codon gives rise to a lysine-to-arginine (K/R) change, which dramatically affects lesion specificity repair by the enzyme [31], while editing at the wobble position is synonymous. The sites of editing are located in a stem-loop structure (Figure 4a and Figure S3 in Additional file 3), where the editing complementary sequence is in the upstream intron 5. Markedly, 221 nucleotides upstream of the K/R site lies a pair of inverted Alus, which are also edited (Figure S3c in Additional file 3). When NEIL1 editing levels were measured in mouse brain, no editing was detected at any of the sites edited in the human transcript (Figure 4b). In addition, no editing was detected for the rat NEIL1 transcript (data not shown), suggesting that editing does not occur in mammals other than primates. To test if editing at this site occurs in other primates and is limited to transcripts containing conserved inverted Alu sequences, we measured editing levels in NEIL1 mRNA from the total brain of another primate, the monkey rhesus macaque. Indeed, the monkey has a highly edited NEIL1 sequence to a similar extent and at the same positions as in the human sequence (Figure 4b). Editing was also detected within the inverted Alu repeats of the rhesus macaque sequence (Figure S3d in Additional file 3).

### Human NEIL1 editing is markedly decreased in the absence of adjacent Alus

To prove that editing of NEIL1 is induced by the upstream Alu elements, we made a *NEIL1* minigene including intron 5 (with the inverted Alu repeats) and exon 6, containing the K/R site of editing (Figure 4a). This construct was used as an editing reporter after co-transfection into HEK293 cells together with an ADAR1 or ADAR2 expression vector. Endogenous editing was also analyzed in HeLa cells. Editing efficiency was measured by Sanger sequencing after RT-PCR on extracted RNA. The human NEIL1 reporter (hNEIL1) was highly edited at the K/R site, showing a dominating G peak in the chromatogram after RT-PCR (Figure 4c and Figure S4 in Additional file 3). The first A (−1 site) and the third A (+1 site) were also edited to a similar extent, as seen in human brain tissue (Figure 4). To analyze the dependence of Alu repeats on editing efficiency, the upstream inverted repeats were deleted (hNEIL1 ΔAlu). Indeed, in ADAR1 co-transfections, the editing efficiency at the K/R site decreased from 77% to 45% (*P* = 3.7 × 10^-6^) in the absence of the inverted Alu repeat (Figure 4c). A dramatic decrease (50%, *P* = 0.003) in editing efficiency was also observed in ADAR2 co-transfections and in endogenous editing in HeLa cells (Figure S4 in Additional file 3) in the absence of the inverted Alu repeats. Furthermore, a decrease in editing was also observed at the neighboring −1 and +1 sites.

The edited AAA codon and its enclosed sequence forming the stem-loop required for editing is highly conserved between mice and humans (Figure S3 in Additional file 3). However, *in vivo,* editing of NEIL1 does not occur in the mouse sequence, probably due to the absence of the upstream Alu stem-loop structure (Figure 4b). We therefore tested if the human inverted Alu sequences in NEIL1 could induce editing in the mouse NEIL1 transcript. A mouse mNEIL1 reporter construct equivalent to the sequence in the human NEIL1 reporter was made and used in co-transfections with the editing expression vectors in HEK293 cells and by using endogenous editing in HeLa cells. The K/R site in mNEIL1 was edited in 22% of the transcripts by over-expressed ADAR1 and 17% after ADAR2 co-transfections (Figure 4c and Figure S4 in Additional file 3). Endogenous editing in HeLa cells gave 8% editing of the mouse NEIL1 reporter at the K/R site. To investigate if the human inverted Alu repeats could induce editing in the mouse sequence, the human NEIL1 Alu repeats were cloned into the mouse NEIL1 construct (mNEIL1 + Alu) in an equivalent position to the human sequence. Indeed, in the presence of the Alu repeats, mouse NEIL1 editing (mNEIL1 + Alu) increased from 22% to almost 48% (*P* = 5.998 × 10^-5^) when co-transfected with ADAR1. A twofold increase (*P* = 0.004) in K/R editing was also observed in co-transfections with ADAR2 and by endogenous editing in HeLa cells (Figure S4 in Additional file 3). This result suggests that the mouse NEIL1 sequence makes a good substrate for editing only in the presence of the Alu inverted repeats.

### The Alu element in NEIL1 increases the local concentration of ADAR

**Figure 5:**
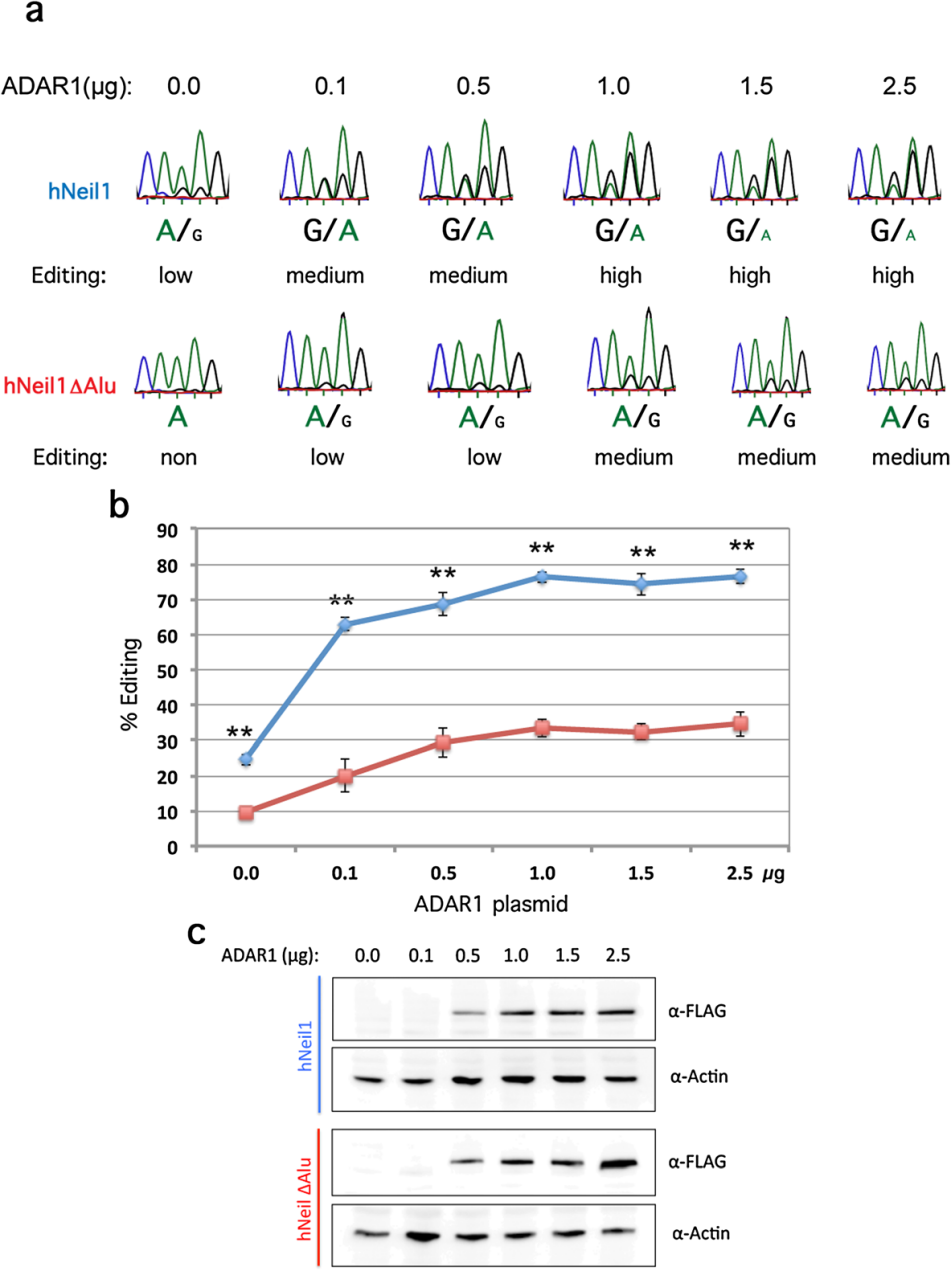
**Titration of ADAR1 co-transfected with hNEIL1 or hNEIL1 ΔAlu in HEK293 cells. (a)** Sequencing chromatograms after RT-PCR from ADAR1 and hNEIL1 or hNEIL1 ΔAlu co-transfections. The reporter constructs were constant (1.5 μg) in each experiment and the concentration of ADAR1 was titrated, ranging from 0 to 2.5 μg. **(b)** Quantification of editing efficiency at the K/R site in hNEIL1 (blue) and hNEIL1 ΔAlu (red) reporters when co-transfected with titrated ADAR1. There were at least triplicates for each concentration. The mean value of the ratio between the A and G peak heights was calculated as percentage editing. Error bars are standard deviation. Significance: **P < 0.05 (two-tailed Student’s *t*-test). **(c)** Western blot analysis of titrated, 0 to 2.5 μg, ADAR1 levels (α-FLAG) during co-transfection with hNEIL1 or hNEIL1 ΔAlu in HEK293 cells. Detection of actin was used as a control for equal loading. RT-PCR, reverse transcription polymerase chain reaction.

Our hypothesis is that a long stem-loop structure forming an almost complete duplex such as the Alu inverted repeats works as a recruitment element of the ADAR proteins. This IE may thereby increase the local concentration of ADAR and facilitate editing at second sites. To determine if this hypothesis holds, we investigated if editing at the K/R site in NEIL1 was less dependent on the concentration of ADAR1 in the presence than in the absence of the Alu IE. We therefore separately transfected a fixed concentration (1.5 μg) of the editing reporters hNEIL1 and hNEIL1 ΔAlu together with titrated concentrations (0 to 2.5 μg) of the ADAR1 expression vector. In theory the substrate containing the Alu IE would require a lower concentration of the editing enzyme to reach a high level of editing than the substrate without the Alu elements. Indeed, already at 0.1 μg of the ADAR1 vector, editing at the K/R site reached a level of 63% in the presence of the Alu inducer, while only 20% (*P* = 0.0001) of the transcripts were edited in the editing reporter lacking the Alu inverted repeats (Figure 5a,b). Editing then reached 76% for the hNEIL1 transcripts at 1 μg of ADAR1, while editing of the hNEIL1 ΔAlu transcripts remained at about 30% (*P* = 5.828 × 10^-5^) despite the addition of 2.5 μg of transfected ADAR1. To ensure that the different amounts of transfected ADAR1 expression vector were proportional to the amount of produced ADAR1 protein, Western blots were made from extracts prepared after the different transfections. As indicated in Figure 5c, the expression of transgenic ADAR1 was proportional to the amount of transfected vector, even though the protein expressed from 0.1 μg of transfected ADAR1 vector could not be detected by Western blot.

The reciprocal experiment was also performed where the hNEIL1 and hNEIL1 ΔAlu editing reporters were titrated (0.1 to 2.5 μg) with a fixed concentration (1 μg) of the ADAR1 expression vector. This gave high levels of edited hNEIL1 transcripts (60%) at low concentrations of the editing reporter. These continued to be efficiently edited even with high concentrations of the editing reporter and with a peak of editing at 66% with 1 μg of the hNEIL1 reporter (Figure S5 in Additional file 3). Editing of the hNEIL1 ΔAlu transcript initiated at 59% (*P* = 0.05) with 0.1 μg of reporter. Editing dramatically decreased when the concentration of the reporter increased to a final editing level of 24% (*P* = 0.0004). This result indicates that in the presence of the Alu IE, a lower concentration of the ADAR enzyme is required to achieve efficient editing at the selective K/R site. It also suggests that the Alu repeats may help in recruiting the editing enzyme to the transcript.

### Human-specific editing of the GLI1 oncogene is Alu dependent

**Figure 6:**
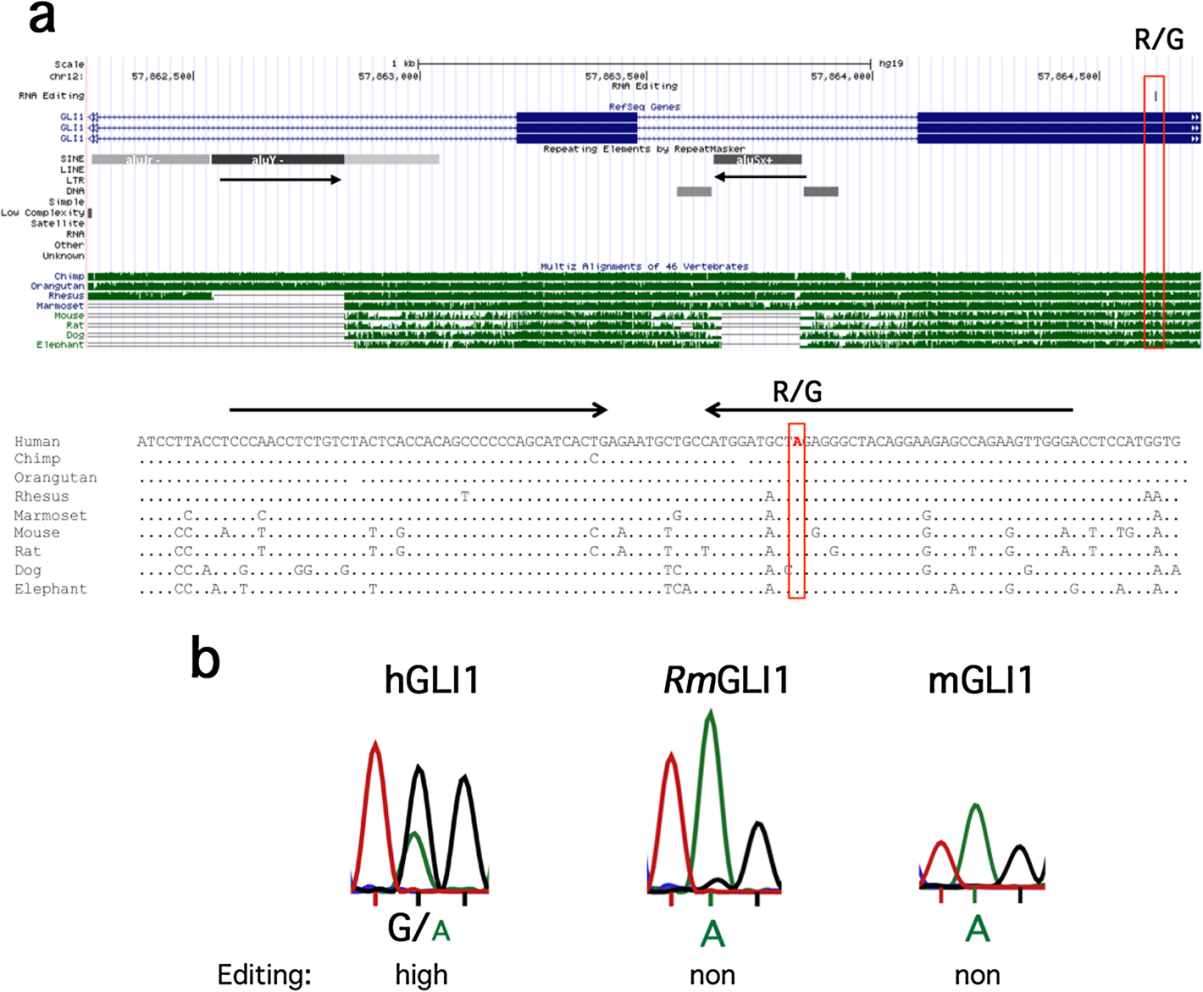
**Inverted Alus and site-selective editing in GLI1. (a)** UCSC genome browser view. Inverted Alu elements are annotated with their family and strandedness in the repetitive elements by RepeatMasker track, and marked with black arrows. While the most downstream alu (aluSx+) is present in all primates, the two upstream alus (aluJr- and aluY-) are absent in some of them (non-apes), as indicated by the Multiz Alignment track. The non-Alu editing site is shown in a red rectangle. Bottom: The sequence flanking the non-Alu editing site. **(b)** Sanger sequencing after RT-PCR on GLI1 transcripts from human (hGLI1), rhesus (RmGLI1) and mouse (mGLI1) brains. Editing was detected as a dual A and G peak in the chromatograms. kb, kilobase; R/G, arginine to glycine; RT-PCR, reverse transcription polymerase chain reaction.

One of the candidate zinc-finger-containing transcription factors detected to contain site-selective editing within encoded sequence proximate to Alu inverted repeats was Glioma-associated oncogene 1 (GLI1). GLI1 act as a transcriptional effector in the Hedgehog (HH) signaling pathway. The transcript of this transcription factor was recently found to be edited in humans, changing an arginine to a glycine (R/G) at position 701 [30,32]. However, very little editing was detected in the mouse GLI1 transcript at the homologous site, even though both sequence and structure are well conserved in the vicinity of the R/G site (Figure 6). This may be due to the lack of an editing IE in the mouse GLI1 transcript. In the human sequence surrounding the edited site, two inverted Alu repeats with the ability to form a stem-loop structure was detected about 780 nucleotides upstream of the R/G site (Figure 6a). These Alu elements are also present in some primates, such as the chimpanzee and orangutan. However, primates more distant from humans, such as the rhesus and marmoset, lack one of the two Alu elements. The rhesus GLI1 transcript is, however, highly conserved in both sequence and RNA structure at the site equivalent to the R/G editing site in humans (Figure 6a). To determine whether the inverted Alus are required for site-selective GLI1 editing, we analyzed the mRNA sequence of GLI1 from rhesus macaque brains. As predicted, editing at the R/G site was very low, close to undetectable (Figure 6b). We therefore conclude that editing may not be detected in some primate species, in spite of a well-conserved sequence surrounding the R/G site, due to the absence of an editing IE.

### ZFP14 editing in the 3′ UTR is human specific and Alu dependent

**Figure 7:**
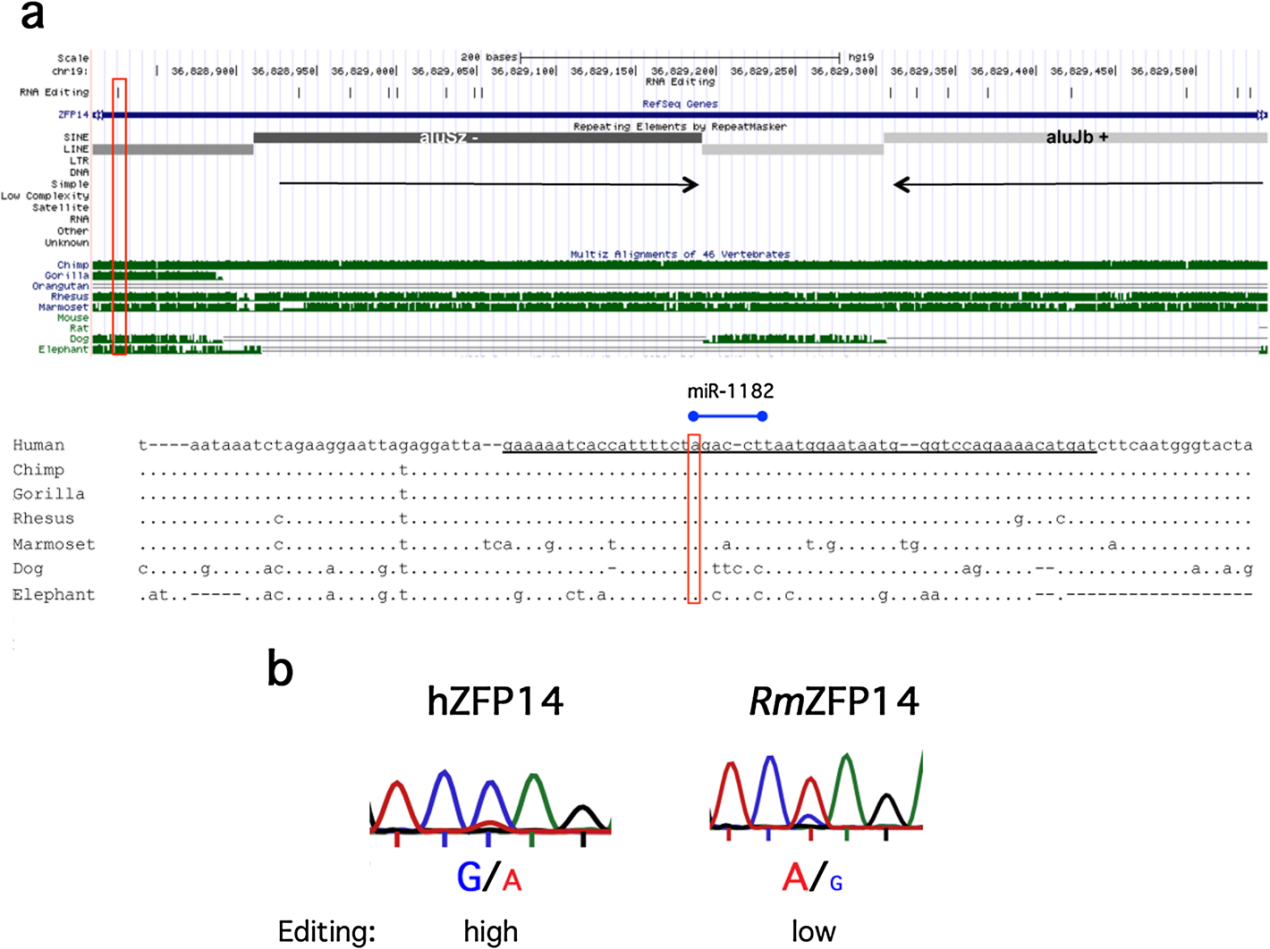
**Inverted Alu elements and site-selective editing in ZFP14. (a)** Top: UCSC genome browser view. Inverted Alu elements are annotated with their family and strandedness in the repetitive elements by RepeatMasker track, and marked with black arrows. **(b)** Reverse strand Sanger sequencing after RT-PCR on ZFP14 transcripts from human (hZFP14) and rhesus (RmZFP14) brain. Editing is detected as a dual A and G peak in the chromatograms.

Another candidate of the edited transcription factor is the Promyelocytic leukemia zinc finger protein, PLZF (also known as ZFP14 and Zbtb16, further referred to as ZFP14). This transcriptional repressor has been shown to regulate major developmental processes, such as spermatogenesis, limb development and hematopoiesis [33]. The transcript has two inverted Alu repeats, which are edited downstream of a non-Alu editing site in the 3′ UTR (Figure 7) [18]. We found that this single editing event has the potential to mask a predicted binding site for miR-1182, a primate-specific microRNA (miRNA). The unedited form of ZFP14 has a perfect match at positions 2 to 8 of miR-1182. The editing site overlaps position 8 of the miRNA. Since this is part of the seed sequence, which is important for target recognition, it is likely to affect the miR-1182 regulation of the ZFP14 transcript. Interestingly miR-1182 has a second predicted binding site in the transcript, which strengthens the prediction that it targets ZFP14. This site is located in one of the Alu elements, 107 nucleotides upstream, where editing is also likely to occur and affect miRNA targeting.

The Alu sequences aluSz and aluJb are present in other primates, such as the chimpanzee, rhesus and marmoset. However, these Alu sequences are not completely conserved among the primate species. Consequently, a putative stem-loop structure formed by the Alus in rhesuses is disrupted by larger and more frequent bulges compared to humans (Figure S6 in Additional file 3). We therefore analyzed the efficiency of editing in the human ZFP14 transcript compared to transcripts from rhesus macaque brains. While the human ZFP14 is edited in the majority of all transcripts, only very low levels of editing could be observed at this site in the rhesus (Figure 7b). Since the sequence of the 3′ UTR is not conserved between primates and other mammals, no editing event could be detected in species other than primates. Taken together this result indicates that editing at this site, with a potential effect on miRNA targeting, is not only primate specific but also restricted to humans or very closely related primates (apes).

## Discussion

In this study we demonstrated that Alu repetitive elements can function as inducers of A-to-I editing in adjacent sequences, affecting the expressed proteome. Alu repeats are primate specific and vary also in abundance within primates. Our observation therefore points to a human- or primate-specific phenomenon that cannot be explained by the sequence at the site of editing.

It has previously been speculated by Li and co-workers that non-Alu A/I editing sites are dependent on nearby edited Alu sequences in the human transcriptome [20]. Their theory was based on the fact the two classes of editing are often found within close proximity in the same transcripts. We were able to confirm their hypothesis, and show that editing in non-repetitive elements often depends on nearby repetitive Alu elements. Our previous analysis showed that induction of site-selective editing at the I/M site of Gabra-3 by a long intronic hairpin structure is independent of editing, and instead depends on the double-strandedness of the IE, indicating that it is ADAR binding rather than editing that induces distant editing [11]. Similarly, inverted Alu repeats appear to be capable of inducing site-selective editing of any substrate with a low basal level of editing. We show here that this is true for the Gabra-3 I/M site, whose endogenous intronic IE can be replaced by inverted Alus, which ‘rescue’ editing (Figure 1b,c). Furthermore, editing of the mouse NEIL1 transcript, endogenously edited to a very low level, is significantly increased by the adjacent incorporation of inverted Alu elements (Figure 4c). Nevertheless editing of the Alu-containing mouse NEIL1 transcript does not reach as high a level of editing efficiency as in the human transcript. Even though the mouse NEIL1 editing substrate can fold into a structure similar to that for humans in the immediate vicinity of the edited site, with a stem-loop of 11 bp, the extension of a possible mouse stem-loop is much shorter than the human stem-loop. This may explain the lower level of editing induction in mice.

We and others have previously suggested that a low basic level of editing detected throughout the transcriptome is a source for adaptive evolution [34,35]. Hairpin structures made by Alu elements that can induce editing at adjacent sites add another dimension to this phenomenon and may confer a selective advantage by giving rise to functional editing events. This further increases genetic variety, since both isoforms (edited and non-edited) can exist in parallel and may play a role in shaping the transcriptomic landscape and the evolution of primates. Since editing is commonly found within transcripts coding for genes expressed in the central nervous system, this may contribute specifically to the complexity, as well as the evolution, of the human brain.

We find a striking enrichment of site-selective editing linked to Alu IEs in transcripts coding for zinc finger proteins. This may be attributed to the enrichment of Alu elements in zinc finger transcription factors that have been detected in primates [29]. Nevertheless, Alu- induced editing of these transcription factors may add to their variability. The repertoire of factors expressed from these genes may then be regulated during development, influenced by environmental conditions such as stress or during an immune response. We specifically show that two of these transcription factors, GLI1 and ZFP14, possess primate-specific editing with functional effects. In the GLI1 transcript, the selective R/G site has previously been shown to be highly edited in humans, while the mouse sequence is not [32]. We confirmed this observation, and also found that despite the high sequence conservation of the stem-loop harboring the R/G site between humans and rhesus monkeys, this site was poorly edited in the monkey (Figure 6b). The difference in editing efficiency can be explained by the absence of the AluY repeat element downstream of the R/G site in the rhesus, which participates in forming the long inverted-Alus stem in humans. The rhesus, therefore, cannot form the editing IE, and thus no induction of editing occurs. Zaphiropoulos and co-workers showed that the R/G change after editing in human GLI1, increases its capacity to activate transcription and makes it less susceptible to inhibition by a HH signaling suppressor [32]. At the same time, GLI1 editing reduces its responsiveness to the Dyrk 1a kinase. These findings clearly show that editing of a transcription factor can affect the expression of significant parts of the transcriptome, which is unique to humans and perhaps closely related primates.

The editing events may not only affect the expression and function of genes. Increasing evidence show that modifications in the 3′ UTR of encoded genes can have dramatic effects on transcript stability, transport and localization of the mature mRNA. The ZFP14 or Plzf transcript encodes for a transcription factor with nine Krüppel-type sequence-specific zinc fingers, and is localized mainly in the nucleus, where it functions predominantly as a transcriptional repressor. Editing of the 3′ UTR in this transcript may prevent processing by miR-1182 and thereby increase stability. Since the 3′ UTR of this gene is only conserved in primates closely related to humans (apes), this mechanism for regulation will only occur in species with a highly developed brain (Figure 7a). Furthermore, since site-selective editing has been shown to be regulated during development with very low levels of editing during embryogenesis, it is likely that the prevention of miR-1182 binding will only occur in the mature animal.

## Conclusions

**Figure 8:**
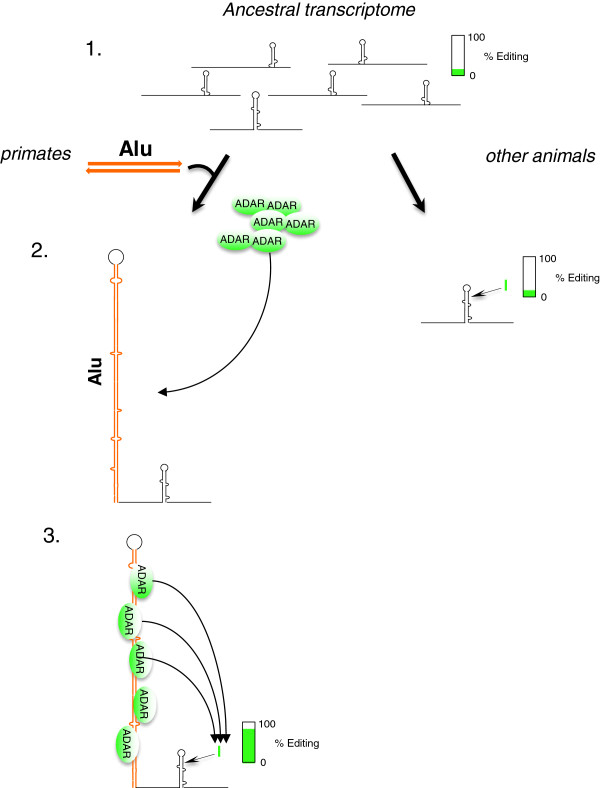
**A model for human- or primate-specific adenosine to inosine editing induced by Alu inverted repeats. (1)** All metazoans have a basic low level of RNA editing in their transcriptome of 1% to 2%. **(2)** Alu elements have been integrated into the primate genome. Pairs of adjacent Alus with inverted orientation, form long and stable duplexes in transcripts, which act as recruitment elements for the ADAR enzymes. **(3)** A high ADAR concentration at the inverted Alus gives high editing efficiency at single sites in nearby short hairpins.

The human transcriptome is exceptionally prone to A-to-I RNA editing. This is mainly explained by the frequently edited inverted Alu repeats, which are unique to primates. The function of editing within these repeats has, however, hitherto largely been unknown. In the present work we propose a model where two primate-specific adjacent Alu repeats function as a recruitment element for the ADAR editing enzymes (Figure 8). The increase in concentration of ADAR enzyme(s) causes induced editing at single sites located several hundred nucleotides away within the same transcript. Taken together, our findings show for the first time that site-selective RNA editing in the brain can be induced by *cis*-acting Alu elements, thereby contributing to primate and human genome evolution by a process that is absent from other species. The primate-specific enrichment of site-selective editing with functional consequences for transcription factors indicates that editing may contribute profoundly to transcriptomic regulation and the variety in transcript isoforms in the primate brain.

## Materials and methods

### Plasmids and substrate mutagenesis

The Gabra-3 editing reporter construct pGARα3-I/M (Gabra-3 WT) was generated from the mouse genome sequence. The ADAR2 expression vector pcDNA3 FLAG/rADAR2 has previously been described [10,36]. The ADAR1 expression vector pCS DRADA-FLIS6 [37] was a kind gift from Mary O’Connell. The deletion mutant (Gabra-3ΔIE) has previously been described [11]. The editing reporters Gabra-3 US IE, DDS IE and Alu-IE were generated by PCR amplification of the mouse Gabra-3 IE sequence. The sequence of the inverted Alus in the 3′ UTR of the human PSMB2 gene was cloned into the Gabra-3ΔIE reporter at different positions (US IE 150 nucleotides upstream of the I/M site, DDS IE 300 nucleotides downstream of the I/M site, Alu-IE 150 nucleotides downstream of I/M site, replacing the IE). Primer sequences were designed with a restriction site overhang and were as follows:

•US IE (BamHI): (FW) 5′-aataaggatccaggaagggctgagaagcacttcc-3′, (RW) 5′-aataaggatccaggccagattaccaagaagc-3′

•DDS IE (NotI): (FW) 5′-aataagcggccgcaggaagggctgagaagcacacttcc-3′, (RW) 5′-aataagcggccgcaggccagattaccaagaagc-3′

•Alu-IE (NheI): (FW) 5′-aatttgctagcgtttcttccatccctataatcc-3′, (RW) 5′-aatttgctagcggtcaagaaccactgttttaatagc-3′

The human NEIL1 and mouse NEIL1 editing reporters were generated by PCR amplification from the genomic *NEIL1* gene, and cloned into pcDNA3 FLAG. Primer sequences were as follows:

•hNEIL1: (FW) 5′-gcccggagctgaccctgagccag-3, (RW) 5′-ggaaccagatggtacggccatgcc-3′

•hNeil ΔAlu: (FW) 5′-ggacaaggattcttaatcccactcc-3′, (RW) as above for hNEIL1

•mouse NEIL1: (FW) 5′-gcaagtttccactttctacc-3′, (RW) 5′-ccagatggtacggccatgccgg-3'

The mouse NEIL1 + Alu was generated by PCR amplification of the human inverted Alus upstream of the K/R site in human NEIL1 using primers with Nhe1 restriction site overhang. The PCR fragment was cloned, using Nhe1, into the mNEIL1 reporter at the position corresponding to the human sequence. Primer sequences were as follows:

•mNEIL1 + Alu (Nhe1): (FW) 5′-aatttgctagcggctgggcgcagtggctcatgc-3′, (RW) 5′-atttgctagctggccctgtgcagtggccacac-3′

Restriction sites were created and also depleted after insertion of the fragments in the Gabra-3ΔIE reporter and in the mNEIL1 reporter using QuickChange site-directed mutagenesis (Stratagene, La Jolla, USA). All mutants were verified by Sanger sequencing (Eurofins MWG Operon, Ebersberg, Germany).

### Transfections

Reporter constructs (1.5 μg) were co-transfected with ADAR1 or ADAR2 (2.5 μg) expression vectors into HEK293 cells and grown in six-well plates. For endogenous editing, the reporter constructs (4 μg) were transfected into HeLa cells. LIPOFECTAMINE™ 2000 (Invitrogen, Carlsbad, USA) was used in all transfections. The transfection efficiency was comparable between separate experiments. Control transfections using an empty vector co-expressed with or without the substrates were done for each experiment. RNA was isolated 48 hr (HEK293) and 72 hr (HeLa) after transfection using GenElute7™ mammalian total RNA isolation (Sigma-Aldrich, St. Louis, USA), and treated with DNase-1 Amplification Grade (Sigma-Aldrich, St. Louis, USA). The cDNA was generated using random hexamer deoxyoligonucleotides and SuperscriptII RT (Invitrogen, Carlsbad, USA). Negative control reactions without reverse transcriptase were performed in all RT-PCR experiments to exclude genomic DNA contamination. The following PCR was made using Taq (Invitrogen, Carlsbad, USA). Primers used for the PCR reactions for Gabra-3 WT and Gabra-3 mutant reporters were: (FW) 5′-ggtgtcaccactgttctcacc-3′ and (RE) 5′-gctgtggatgtaataagactcc-3′. Primers used for the PCR reactions of human NEIL1 and mouse NEIL1 reporters were as described above.

### Analysis of RNA editing *in vivo*

Experiments were carried out on tissue extracted from an adult NMRI mouse (whole brain) and a human (cerebellum). The experiments on mouse brain tissue were approved by Stockholms Norra Djurförsöksetiska nämnd and have permission number: Dnr N 410/12. The human brain tissue was provided by the Huddinge Brain Bank and the experiments were approved by the Karolinska Institute, Forskningskommité Syd, with the permission number: Dnr 84/02. Total RNA was isolated using the TRIZol reagent protocol (Invitrogen, Carlsbad, USA) and treated with DNase-1 Amplification Grade (Sigma-Aldrich, St. Louis, USA). Genomic human and mouse DNA from the same individuals were purified using QIAamp DNA Mini Kit (Qiagen, Hilden, Germany). DNase1-treated total RNA and genomic DNA from the brain of an adult rhesus monkey was purchased (Zyagen, San Diego, USA). First strand cDNA was synthesized with Superscript II and random hexamer deoxyoligonucleotides (Invitrogen, Carlsbad, USA). Negative control reactions without reverse transcriptase were performed in all RT-PCR experiments to exclude genomic DNA contamination. A standard PCR protocol was used for NEIL1 and ZFP14 and nested PCR was performed to amplify GLI1 (initial PCR 20 cycles, nested PCR 25 cycles). Primers used for the PCR reactions were as follows:

•human NEIL1: (FW) 5′-gcccggagctgaccctgagccag-3′, (RW) 5′- ggaaccagatggtacggccatgcc-3′

•mouse NEIL1: (FW) 5′-agcccagagctgaccctgagccag-3′, (RW) 5′- ccagatggtacggccatgccgg-3′

•rhesus NEIL1: (FW) 5′-gcctgaagctgaccctcagccag-3′, (RW) 5-′ggaaccagatggtacggccatgcc-3′

•human GLI1: (FW initial) 5′-gcagccaatacagacagtggtg-3′, (FW nested) 5′-ccagtgacccagcccaggctg-3′, (RW initial and nested) 5′-ggtggaacctacagccagtgtcc-3′

•rhesus GLI1: (FW initial) 5′-tgtcaagacagtgcatggtcctg-3′, (FW nested) 5′-gctccagctagagctcagagg-3′, (RW initial and nested) 5′-ctgtaggctccacctagagcc-3′

•mouse GLI1: (FW initial) 5′-acacgtgaagacagtgcatg-3′, (FW nested) 5′-gttcaagagcctgggatgtg-3′, (RW initial and nested) 5′-gacactggctataggcagcac-3′

•human ZFP14: (FW) 5′-gaagaagtctaataaatctag-3′, (RW) 5′-ccatcagtggaggatcctggaacc-3′

•rhesus ZFP14: (FW) 5′-gaagtctaataaatccagaagg-3′, (RW) 5′-acactgttcatctagtcccc-3′

The PCR products were gel-purified using NucleoSpin Gel and PCR Clean-up (Macherey-Nagel, Düren, Germany) and editing was determined by Sanger sequencing (Eurofins MWG Operon, Ebersberg, Germany).

### Calculation of editing frequency

To evaluate the level of edited transcripts, RNA from at least three independent experiments was sequenced. Editing was determined by measuring the ratio between the A peak and the G peak heights in individual chromatograms using FinchTV. Percentage editing was calculated as the peak height of G/(A + G) × 100. *P* values were calculated using a two-tailed Student’s *t*-test. In addition, percentage editing at the I/M site in the Gabra-3 transcript was compared to editing determined by 454 high-throughput sequencing as in [11], where the levels of edited transcripts were grouped from *non* to *full* in five stages (non: <10%, low: 10% to 25%, medium: 25% to 50%, high: 50% to 75%, full: 75% to 100%) according to the mean value of the G peak derived from triplicates of the experiments.

### Western blot

Whole-cell extracts of transfected HEK293 cells were prepared using Lysis-M (Roche, Mannheim, Germany), supplemented with protease inhibitor cocktail (Roche, Mannheim, Germany). Samples for Western blot were boiled for 10 minutes with a Laemmli sample buffer containing β-mercaptoethanol prior to fractionation by electrophoresis in 10% polyacrylamide gels and transfer to a polyvinyl diflouride (PVDF) membrane. Membranes were probed with α-FLAG (1:1000) or α-actin (1:250), both from Sigma-Aldrich, St. Louis, MO, USA. Horseradish peroxidase-conjugated anti-rabbit IgG (Dako, Glostrup, Denmark) was used as secondary antibodies. Blots were developed using the WesternBright Sirius chemiluminescence detection system (Advansta, Menlo Park, CA, USA).

### Prediction of RNA secondary structure

RNA secondary structure predictions were made using Mfold [38] and Sfold [39]. All secondary structures mentioned were observed by both algorithms.

### Editing dataset compilation and characterization

Editing sites for analysis were compiled from several transcriptome-wide screenings, summarized in Table S1 in Additional file 1. Edited Alus were selected for analysis because they bear direct evidence for ADAR recruitment. Only sites on RefSeq transcripts were included, and only those identified in tissues (rather than immortalized cells), to minimize the likelihood of false positives or experimental artifacts. Variant Effect Predictor (Ensembl, [40]) was used to annotate editing sites with genomic element categories (RefSeq hg19 annotations), and to identify potential effects of editing on the genes. Multiple annotations are shown where the editing site was located on multiple genomic elements. The random adenosine data were used to generate an expected random distribution of adenosine substitution effects. In addition, since gene-region coverage is often biased in RNA sequencing data (typically against introns), we used the coverage ratios from Ramaswami *et al*. [20], which is also the major source for the data in our analysis, as a rough indicator for correcting the expected distribution for the differential coverage.

### Conservation analysis

For each non-clustered analyzed editing site (missense and UTR sites), genomic intervals of 30 flanking nucleotides were obtained, 15 from each side. Non-exonic nucleotides were removed from the intervals, which extended beyond the exon ends, to maintain uniformity in the compared genomic elements. For 46 vertebrates, PhastCons and PhyloP scores were downloaded for the nucleotides in the genomic intervals from the UCSC table browser [41] and were averaged. The average scores were subsequently used for comparisons between sites proximal (= < 1 kb) and distal (>1 kb) to edited Alus.

### Distance analysis

To obtain random adenosines, genomic SNP coordinates, where adenosine is the reference allele, were downloaded from SNPdb [42] via the UCSC table browser [41]. Only SNPs on RefSeq genes were included, and those overlapping Alu elements were removed by the BedTools suite [43]. Of the remaining SNP coordinates, 20,000 were randomly selected using the random-sort option in the GNU core utilities Sort program [44]. BedTools was then used to separate between Alu and non-Alu editing sites, and to calculate the distances between editing sites and Alus, and random adenosines and Alus.

### Gene ontology and pathway analyses

Gene ontology and pathway analyses were performed by DAVID [27,28]. The parameters used for the functional annotation chart were a minimum count of 3 and ease of 0.1. For the annotation clustering, the same minimum count was used, with an ease of 0.01. Statistical tests were performed by SPSS 17.0 (SPSS, Chicago, IL, USA) and R [45]. The normality of the data distribution was assessed based on Q-Q plots. Levene’s test was used to examine equality of variance. Plots and graphs were made by R and Microsoft Excel (Microsoft Ltd, Albuquerque, USA). The 3’UTR of ZFP14 was scanned for microRNA targets using PITA [46], using a minimum of seven matches to the seed sequence and one wobble site.

## Abbreviations

A-to-I: adenosine to inosine; bp: base pair; DDS IE: downstream inducer element; dsRNA: double-stranded RNA; I/M: isoleucine to methionine; IE: inducer element; K/R: lysine-to-arginine; kb: kilobase; miRNA: microRNA; PCR: polymerase chain reaction; R/G: arginine to glycine; RT-PCR: reverse transcription polymerase chain reaction; SNP: single nucleotide polymorphism; US IE: upstream inducer element; UTR: untranslated region; WT: wild-type; ΔIE: deleted inducer element.

## Competing interests

The authors declare that they have no competing interests.

## Authors' contributions

CD and MÖ designed the experiments. CD performed the molecular genetic studies. MB performed the Western blot analysis. GS designed and performed the computational analyses. CD, GS and MÖ wrote the manuscript. All authors read and approved the final manuscript.

## Supplementary Material

Additional file 1: Table S1 Sources of editing sites and edited Alus compiled from literature mining. Click here for file

Additional file 2: Table S2 All non-Alu editing sites included in the study. Click here for file

Additional file 3: Figure S1 Distance distribution between edited sites and nearest edited Alu, after exclusion of clustered sites. Positive/negative distance values indicate that the edited Alu is downstream/upstream of the (non-Alu) editing site, respectively. Green bars: distance from editing sites to the edited Alus. Red bars: distance from random adenosines to edited Alus. After removal of clustered sites, the frequency of sites closest to edited Alus increased, as did the preference for the Alus to be downstream of the edited site (compare with Figure 2c). **Figure S2.** Mean PhyloP and PhastCons conservation scores of sequences flanking UTR editing sites proximal to (<=1 kb), and distal to (>1 kb) edited Alu. **Figure S3.** NEIL1 sequence alignment of selected placental mammals. **Figure S4.** Editing of the human NEIL1 transcript in the presence and absence of the upstream Alu elements. **Figure S5.** Titration of hNEIL1 or hNEIL1 ΔAlu reporters co-transfected with a constant concentration of ADAR1 expression vector of 1.5 μg into HEK293 cells. **Figure S6.** Predicted RNA secondary structures of the inverted Alu repeats in the human and Rhesus ZFP14 transcripts, as presented in Figure 7. Click here for file

Additional file 4: Table S3 Ultra-conserved sequences flanking Alu-independent editing sites. Click here for file

Additional file 5: Table S4 All significantly enriched terms and genes with editing sites close to inverted Alu repeats. Click here for file

Additional file 6: Table S5 Clusters of annotation enriched among genes containing non-Alu editing sites adjacent to edited Alus. Click here for file
