# Supplementary material for: Alu elements shape the primate transcriptome by cis-regulation of RNA editing
Source: Genome Biol. 2014 Feb 3;15(2):R28. doi: 10.1186/gb-2014-15-2-r28 (PMC4053975; doi:10.1186/gb-2014-15-2-r28)
Supplement: Additional file 1: Table S1 — Sources of editing sites and edited Alus compiled from literature mining. [file gb-2014-15-2-r28-S1.docx]

| **Source** | **Edited Alus** | **Non-Alu editing sites** |
| --- | --- | --- |
| Ramaswami G et al. 2012: (YH dataset) | 106,097 | 7,222 |
| Carmi et al. 2011 - hyper_Ed Alus | 258 | - |
| Carmi et al. 2011 - ultra_Ed Alus | 755 | - |
| Kiran & Baranov, 2010 | 6,272 | 3,537 |
| Peng et al. 2012 | 5,385 | 1,105 |
| Total unique elements | 108,838 (19.1%) | 10,650 |

**Supplementary Table 1.** Sources of editing sites and edited Alus compiled from literature mining.
