# Supplementary material for: Alu elements shape the primate transcriptome by cis-regulation of RNA editing
Source: Genome Biol. 2014 Feb 3;15(2):R28. doi: 10.1186/gb-2014-15-2-r28 (PMC4053975; doi:10.1186/gb-2014-15-2-r28)
Supplement: Additional file 3: Figure S1 — Distance distribution between edited sites and nearest edited Alu, after exclusion of clustered sites. Positive/negative distance values indicate that the edited Alu is downstream/upstream of the (non-Alu) editing site, respectively. Green bars: distance from editing sites to the edited Alus. Red bars: distance from random adenosines to edited Alus. After removal of clustered sites, the frequency of sites closest to edited Alus increased, as did the preference for the Alus to be downstream of the edited site (compare with Figure 2c). Figure S2. Mean PhyloP and PhastCons conservation scores of sequences flanking UTR editing sites proximal to (<=1 kb), and distal to (>1 kb) edited Alu. Figure S3. NEIL1 sequence alignment of selected placental mammals. Figure S4. Editing of the human NEIL1 transcript in the presence and absence of the upstream Alu elements. Figure S5. Titration of hNEIL1 or hNEIL1 ΔAlu reporters co-transfected with a constant concentration of ADAR1 expression vector of 1.5 μg into HEK293 cells. Figure S6. Predicted RNA secondary structures of the inverted Alu repeats in the human and Rhesus ZFP14 transcripts, as presented in Figure 7. [file gb-2014-15-2-r28-S3.pdf]

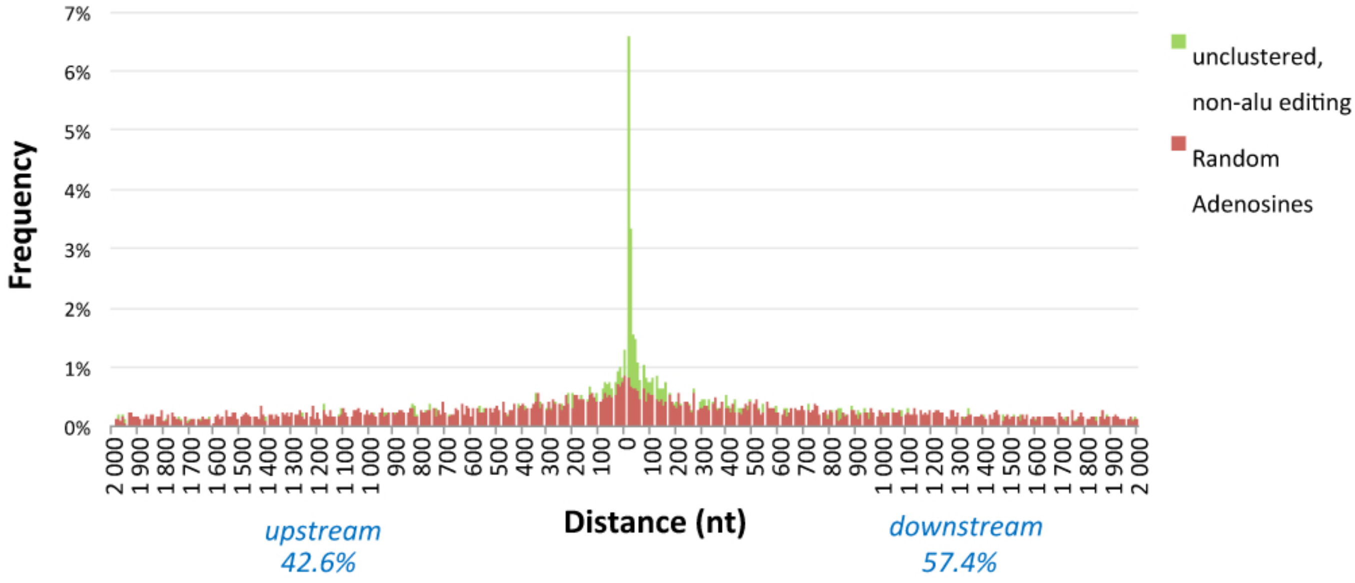

Supplementary Figure 1. Distance distribution plot between edited sites and nearest edited Alu, after exclusion of clustered sites. Positive/negative distance values are indicate that the edited Alu id downstream/upstream of the (non-Alu) editing site, respectively. Green bars: distance from editing sites to the edited Alus. Red bars: distance from random adenosines to edited Alus. After removal of clustered sites, the frequency of sites closest to edited Alus increased, as well as the preference for the Alus to be downstream of the edited site (compare with Figure 2c).

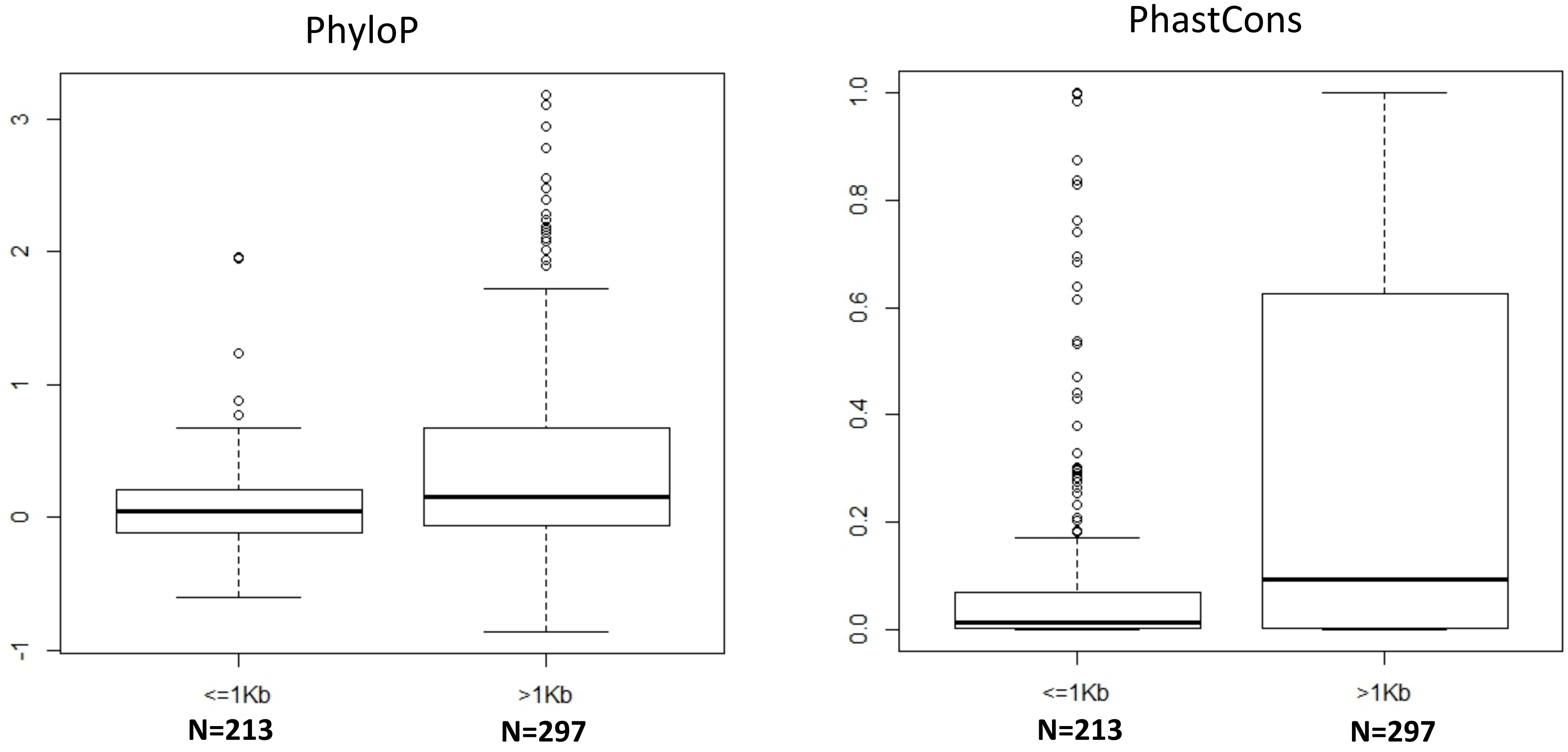

Supplementary Figure 2. Mean PhyloP and PhastCons conservation scores of sequences flanking UTR editing sites proximal to (<=1kb), and distal to (>1kb) edited alu. Conservation scores of the editing site, along with15 nucleotides upstream and downstream were averaged. Distal sites are significantly more conserved (T-test $p=4.8x10^{-11}$, Mann-Whitney U-test $p=1.1x10^{-6}$ for PhyloP and PhastCons respectively).

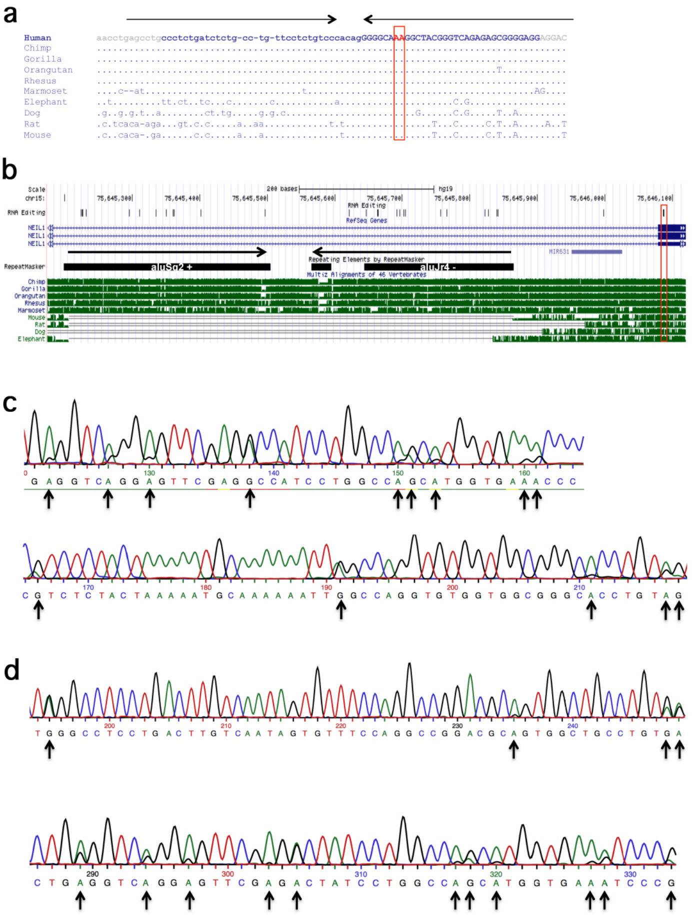

Supplementary Figure 3. Neil1 sequense alingment of selected placental mammals. **(a)** Conservation of sequences at the stem loop structure including the K/R editing site. **(b)** Conservation of the Alu repeat sequences upstream of the K/R site. Arrows indicate complememntary inverted sequences. Boxed nucleotides highlighted in red are edited adenosines at the K/R site. **(c)** Indicated edited adenosines (arrows) within the Alu repeats in the human Neil1 transcript detected by Sanger sequencing after RT-PCR. **(d)** Edited adenosines (arrows) within the Alu repeats in the Neil1 transcript from *Rhesus macaque.*

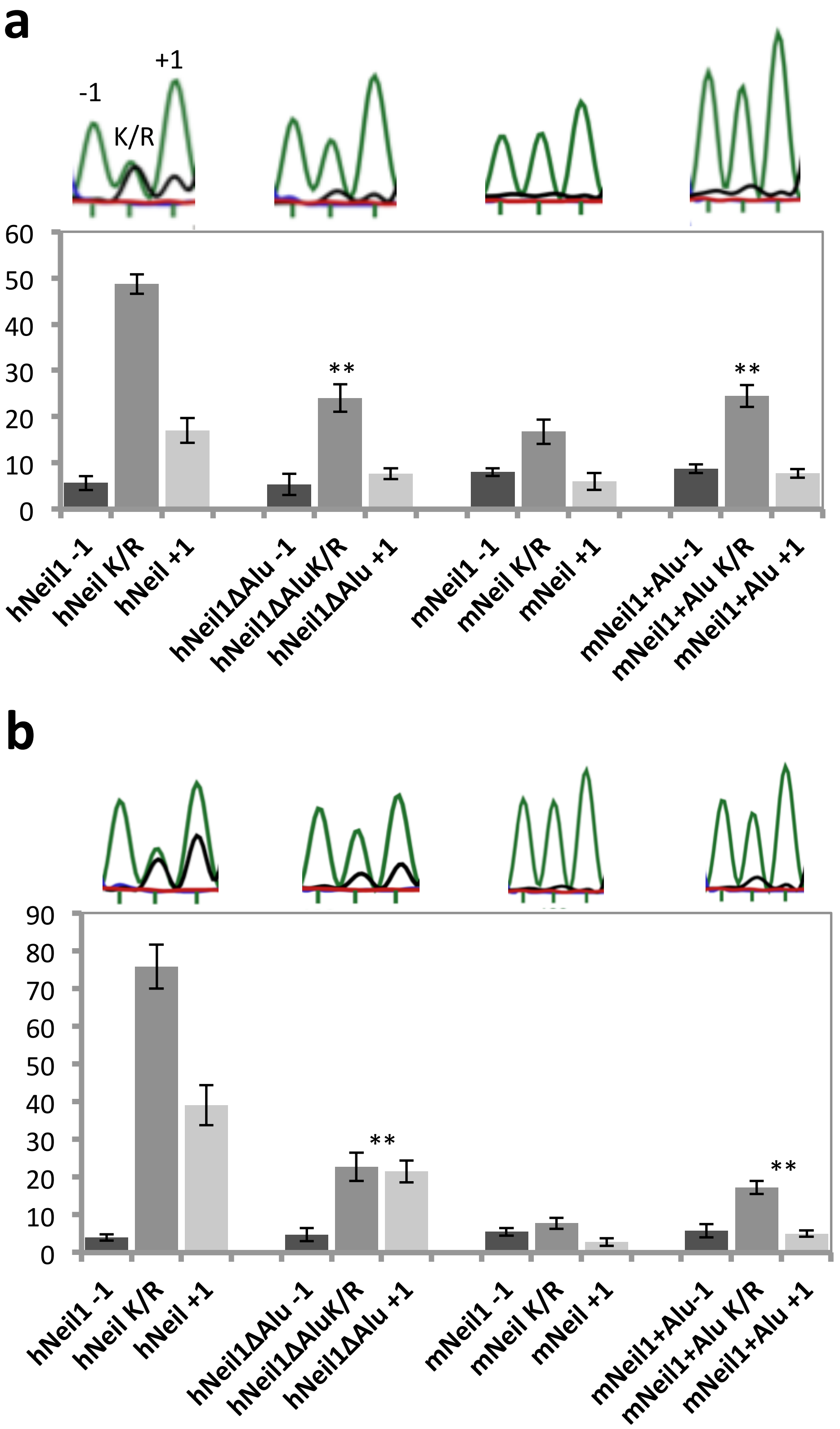

Supplementary Figure 4. Editing of the human Neil1 transcript in presence and absence of the upstream Alu elements. **(a)** Sanger sequencing after RT-PCR on RNA from the different Neil1 constructs co-transfected with ADAR2 into HEK293 cells. Below, quantification of the editing efficiency at the adjacent -1, K/R and +1 sites. **(b)** Sanger sequencing on RNA from the different Neil1 constructs after endogenous editing in HeLa cells. Below, quantification of editing efficiency. The mean value of the ratio between the A and G peak heights from at least three individual experiments were calculated and presented as percent editing (for details see methods section). Error bars are standard deviation and significance is indicated, ** $p < 0.05$ (two-tailed student´s t-test).

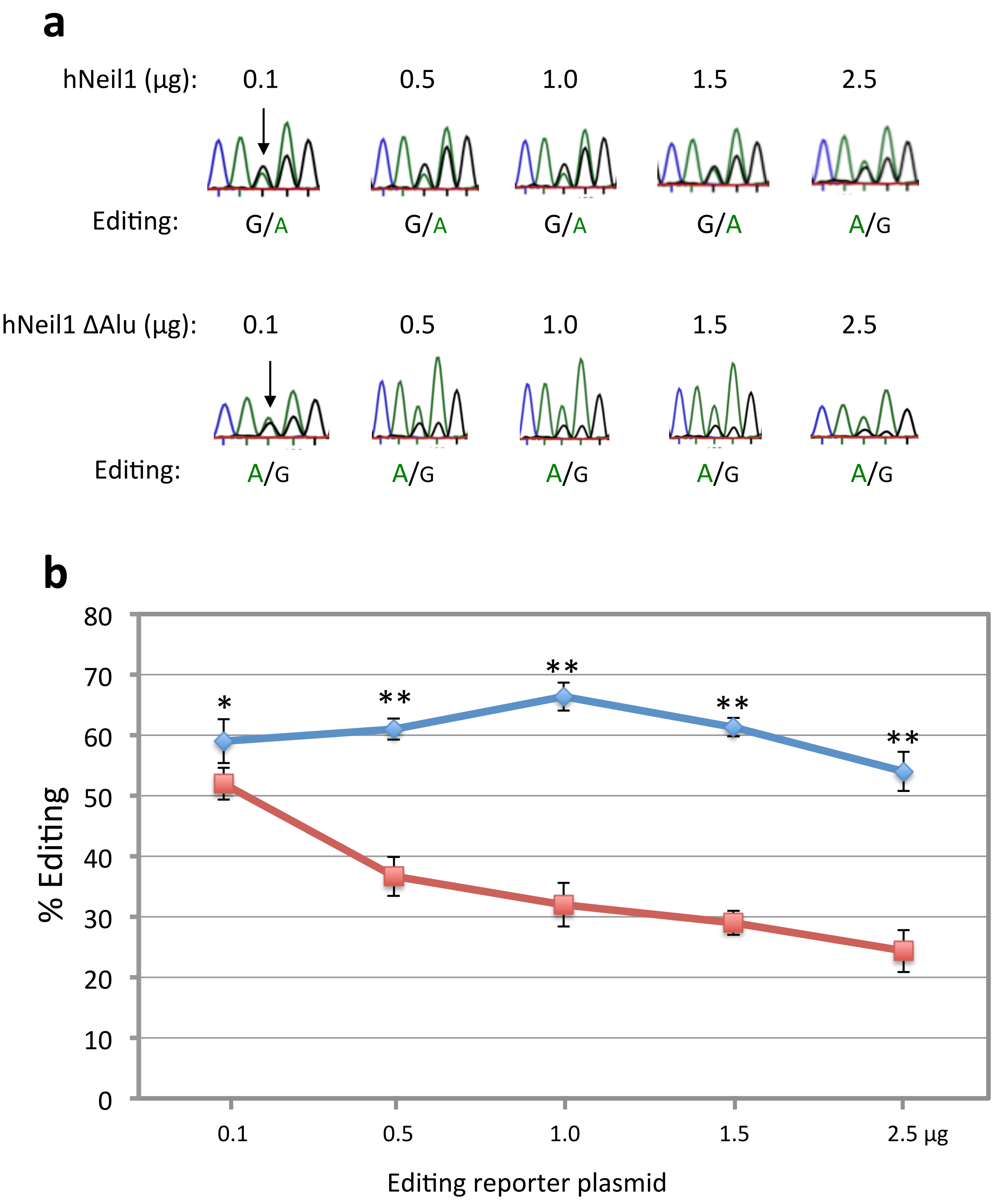

Supplementary Figure 5. Titration of hNeil1 or hNeil1 ΔAlu reporters cotransfected with a constant concentration of ADAR1 expression vector of 1.5 µg into HEK293 cells. **(a)** Sequencing chromatograms after RT-PCR on RNA from the reporter constructs. Arrows indicates the position of editing at the K/R site. **(b)** Quantification of editing efficiency at the K/R site in hNeil (blue) and hNeil1ΔAlu (red) reporters when co-transfected with ADAR1. A minimum of triplicates were done for each concentration and the mean value of the ratio between the A and G peak heights were calculated and presented as percent editing. Error bars are standard deviation and significance is indicated * $p = 0.05$, ** $p < 0.05$ (two-tailed student´s t-test).

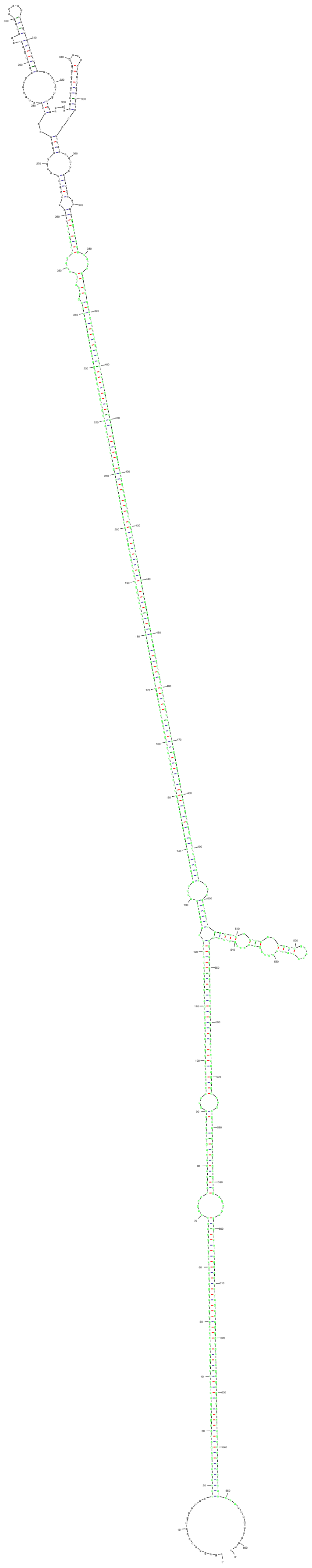

Human ZFP14 Alus

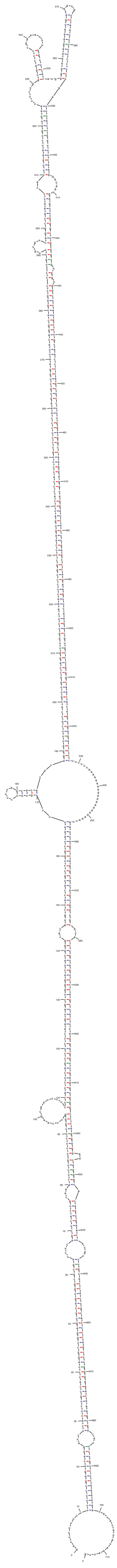

Rhesus ZFP14 Alus

Supplementary Figure 6. Predicted Mfold RNA secondary structures of the Alu inverted repeats in the human and Rhesus ZFP14 transcript, as presented in figure 7.
